# Supplementary material for: Establishment of Immune Biobank for Vaccine Immunogenicity Prediction Using In Vitro and In Silico Methods Against Porcine Reproductive and Respiratory Syndrome Virus
Source: Vaccines (Basel). 2025 Oct 14;13(10):1052. doi: 10.3390/vaccines13101052 (PMC12567839; doi:10.3390/vaccines13101052)
Supplement: Supplementary file 1 [file vaccines-13-01052-s001.zip › vaccines-3891876-supplementary.pdf]

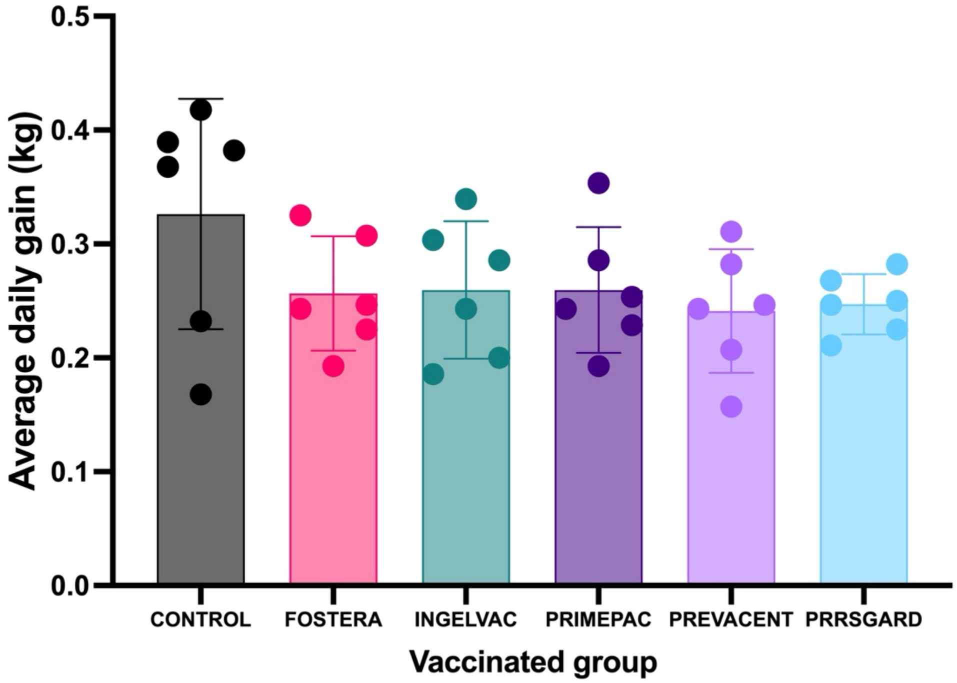

Figure S1. Average daily gain (ADG) at 28 dpv in vaccinated and control pigs.

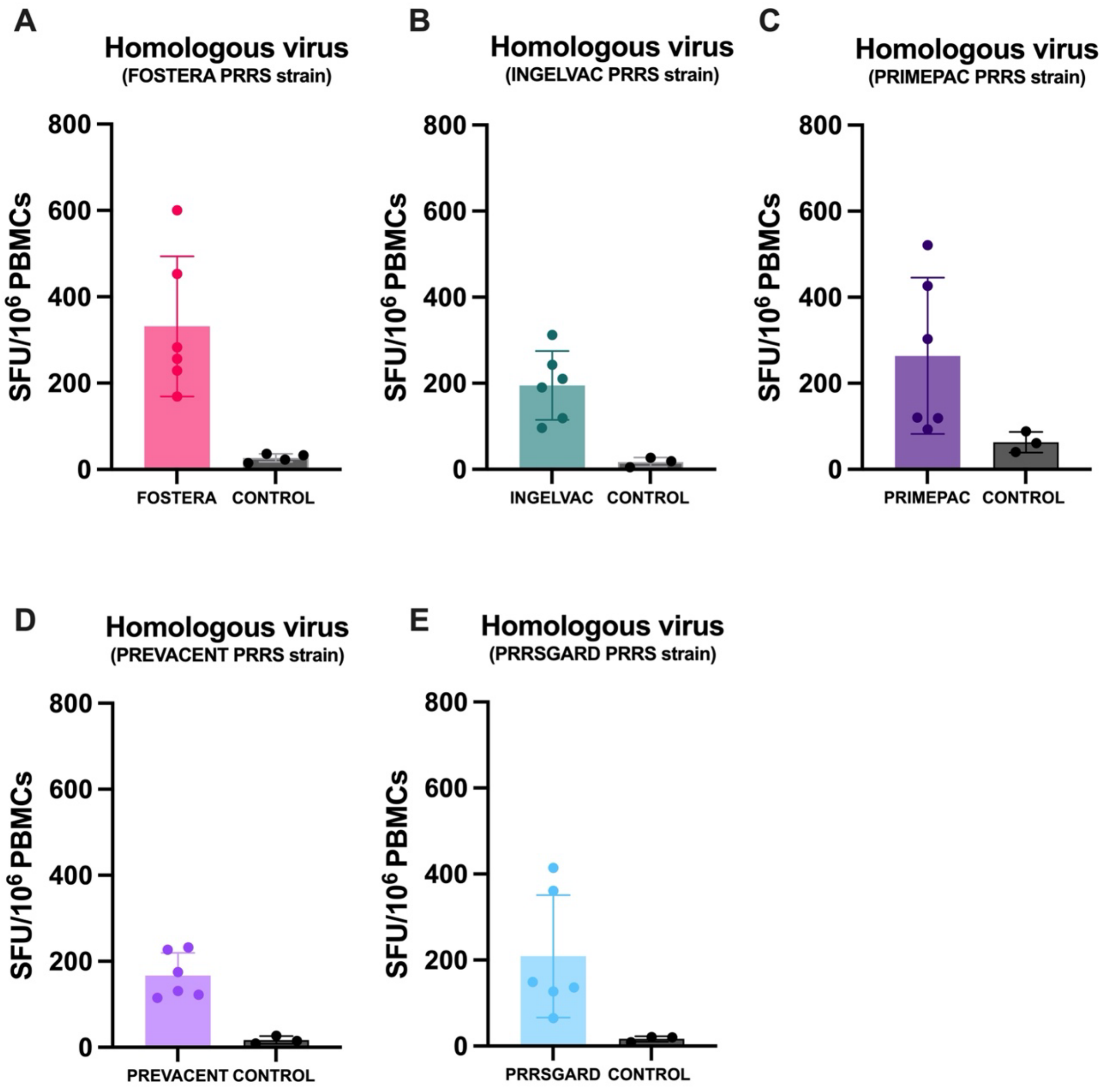

Figure S2. PRRSV-specific IFNγ–producing cells following homologous restimulation with vaccine strains: (A) Fostera, (B) Ingelvac, (C) PrimePac, (D) Prevacent, and (E) PRRSGard.

Table S1. The nucleotide and amino acid similarities based on complete genome sequences between vaccine strains and different PRRSV strains used in this study.

Strains (Classification)	Accession No.	Similarities (%)	Type of vaccines				
			Fostera™ PRRS MLV	Ingelvac® PRRS MLV	PrimePac® PRRS	Prevacent™ PRRS	PRRSGard®
VR2332 (L5A.1)	U87392	Nucleotide similarity	90.68%	99.79%	93.18%	83.77%	92.61%
		Amino acid similarity	76.86%	99.53%	84.02%	61.19%	82.26%
NC134 (L1C.3)	ON844087	Nucleotide similarity	82.89%	83.40%	82.49%	80.78%	83.44%
		Amino acid similarity	58.76%	60.45%	58.00%	53.89%	60.48%
NC20-1 (L1A.17)	OR805486	Nucleotide similarity	81.43%	81.51%	81.25%	80.99%	81.41%
		Amino acid similarity	55.89%	55.91%	55.25%	55.11%	55.98%
NC23-11 (L1A-unclassified)	PP658207	Nucleotide similarity	81.47%	81.36%	81.17%	80.72%	81.50%
		Amino acid similarity	55.88%	55.89%	55.23%	54.61%	56.19%
NC24-6 (L1C.5)	PQ871205	Nucleotide similarity	81.92%	82.32%	81.49%	80.21%	81.87%
		Amino acid similarity	56.55%	57.91%	55.64%	52.93%	56.88%
NC24-9 (L1A-unclassified)	PQ871206	Nucleotide similarity	82.26%	82.11%	81.89%	81.34%	82.29%
		Amino acid similarity	57.66%	57.37%	56.79%	56.09%	57.97%
